# The “opinion matching effect” (OME): A subtle but powerful new form of influence that is apparently being used on the internet

**DOI:** 10.1371/journal.pone.0309897

**Published:** 2024-09-12

**Authors:** Robert Epstein, Yunyi Huang, Miles Megerdoomian, Vanessa R. Zankich

**Affiliations:** American Institute for Behavioral Research and Technology, Vista, CA, United States of America; Academia de Studii Economice din Bucuresti, ROMANIA

## Abstract

In recent years, powerful new forms of influence have been discovered that the internet has made possible. In the present paper, we introduce another new form of influence which we call the “opinion matching effect” (OME). Many websites now promise to help people form opinions about products, political candidates, and political parties by first administering a short quiz and then informing people how closely their answers match product characteristics or the views of a candidate or party. But what if the matching algorithm is biased? We first present data from real opinion matching websites, showing that responding at random to their online quizzes can produce significantly higher proportions of recommendations for one political party or ideology than one would expect by chance. We then describe a randomized, controlled, counterbalanced, double-blind experiment that measured the possible impact of this type of matching on the voting preferences of real, undecided voters. With data obtained from a politically diverse sample of 773 eligible US voters, we observed substantial shifts in voting preferences toward our quiz’s favored candidate–between 51% and 95% of the number of people who had supported that candidate before we administered and scored the quiz. These shifts occurred without any participants showing any awareness of having been manipulated. In summary, in the present study we show not only that OME is a large effect; we also show that biased online questionnaires exist that might be shifting people’s opinions without their knowledge.

## 1. Introduction

As human communities grew in size from small tribes into vast cities and countries, leaders have had to develop increasingly effective ways of controlling the thinking and behavior of increasingly larger groups of people. By the early 1900s, social engineering began to progress from mere art to calculated science, beginning, perhaps, with theories of propaganda advanced by Georgy Plekhanov [1] and other early Marxists. The assertion that governments were not only responsible for controlling the masses but that they could use systematic, powerful methods to do so blossomed in capitalist America with the work of Edward L. Bernays [2], often known as the father of public relations. Bernays insisted that experts who master the emerging new techniques of control could be even more powerful than the government itself, constituting “an invisible government which is the true ruling power of our country” [2].

In 1957, journalist Vance Packard published a landmark book called *The Hidden Persuaders* [3], in which he revealed how both companies and politicians had begun working closely with social scientists to develop increasingly powerful ways of manipulating consumers and voters, often employing methods that left people unaware that they were being manipulated. These methods were being developed and tested using controlled experiments; behavioral science was now an essential tool of the marketing professional. Note, however, that these new techniques of control were not necessarily a threat to humanity or democracy, mainly because they were inherently competitive. Every company and politician could use them, but so could their competitors.

In 1961, in President Eisenhower’s farewell speech as president, he warned not only about the rise of a “military-industrial complex,” he also expressed concern about the possible emergence of a “technological elite” that could someday control public policy without people knowing. Such new forces of control could be held in check, said Eisenhower, only by “an alert and knowledgeable citizenry” [4]. Has the public been alert, and are we knowledgeable about new forms of influence that may have come into being in the decades since Eisenhower’s warning? And are these new techniques of control competitive (and therefore relatively benign in aggregate), or do they pose unprecedented threats to democracy and human autonomy?

The rapid proliferation of internet access over the past two decades has in fact created new and especially impactful methods for controlling people’s thinking and behavior, and because internet activity is dominated by a small number of worldwide monopolies–mainly Google and Meta/Facebook–when these new methods of influence are deployed, there appears to be no way to counteract them. If Candidate A posts an attack video online or on television, Candidate B can do the same. But if one of the large online platforms uses subtle techniques to support one candidate, the opposing candidate has no way to counteract that support.

Our research team has discovered, studied, and quantified several of these new methods of influence over the past decade [5–9]. In the present paper, we introduce a new form of online influence we call the opinion matching effect (OME). We first present data showing that the effect has likely been deployed to some extent on the internet, and we then present a controlled experiment that demonstrates the potential power of this effect to shift opinions and voting preferences. Unlike other effects we have studied, OME is not exclusively in the hands of large tech monopolies. We believe, in fact, that it is being used competitively, which means–at the moment, anyway–that it does not pose a serious threat to democracy or human autonomy. That said, if this technique were to be adopted by a large tech monopoly at some point, it would likely have an outsized impact on online users–one that might be difficult or impossible for competitors to counteract. Search engines and social media platforms could also exercise the power they have to promote some opinion matching websites over others.

### 1.1 Invisible influence

A relatively large scientific literature now exists that examines ways of influencing people without their knowledge, and it is beyond the scope of this paper to review that literature in detail. We will describe some salient examples, however.

*The Hidden Persuaders*, the book by Vance Packard we mentioned earlier, was first published in 1957 and is still in print more than 60 years later. It shocked the American public by revealing the surprising extent to which companies and political candidates were collaborating with social scientists to develop new, largely invisible, methods for influencing consumers and voters [3]. Packard noted, for example, that the slow music that many stores were now broadcasting from speakers in their ceilings caused people to walk slower and, in so doing, to make more purchases. This manipulation produced no awareness on the part of consumers, needless to say. (This technique is used to this day, as the reader will likely observe on his or her next visit to a large store [10,11]). Packard described dozens of techniques like this, almost all of which were supported by controlled studies performed by social scientists.

The recent best-selling books *Nudge*, by behavioral economists Richard Thaler and Cass Sunstein [12], and *Sway*, by business author Ori Brafman and psychologist Rom Brafman [13], summarize more recent studies of this sort, and so do two more recent scholarly books, each entitled *Invisible Influence* [14,15]. In one of the studies mentioned in these books, researchers showed that people more often cleaned their eating environments when the subtle odor of a disinfectant cleaner was present than when it was absent [16]. Manipulations of this sort are especially problematic because they often lead people to believe that they are thinking independently–that they have made up their own mind [17,18]. Thaler and Sunstein argue that when unseen forces are guiding people’s behavior, they have lost their freedom. Because no cages and whips are visible, however, they might still feel free and thus not take steps to regain their actual freedom. A number of recent authors have expressed concern about a growing number of invisible manipulations that the internet has made possible, applying terms such as “digital nudging” and “hypernudging” to the new techniques [19,20].

### 1.2 Recommender systems

OME can be considered a special case of recommender systems [21], which have been widely studied in recent years. Controlled studies have shown that computer-generated recommendations impact purchase preferences even when those recommendations are generated randomly [22,23]. The power of such systems is no secret, and they impact more than just purchases. A 2015 study by employees at Netflix concluded, among other things, that the company’s recommender algorithm accounted for “about 80% of hours streamed at Netflix” [24]. In 2018, Neal Mohan, then Chief Product Officer at YouTube, revealed that 70% of the time people spend watching videos on YouTube, they are viewing content recommended by YouTube’s recommender algorithms [25,cf. 26,27]. It has been estimated that 35% of Amazon’s online sales are driven by Amazon’s recommender algorithms [28,cf. 29]. Public officials have expressed particular concern over the company’s practice of ranking Amazon-branded products ahead of competitors’ products in the product lists shown to potential buyers–the equivalent of search results in a search engine [30,31].

Sometimes relatively organic and benign online content can shift thinking and behavior. Online reviews of consumer products posted by legitimate reviewers–actual users of those products who post blogs or YouTube videos, for example–might recommend a product because they genuinely like it, and online product reviews have been shown to impact consumer purchases [32–35]. Because such reviews are inherently competitive, they pose no great threat to consumers, in our view. We are using the term “recommender *systems*,” however, to refer to algorithmically driven content that might influence large numbers of people and that cannot easily be countered either by consumers or competing businesses. When marketers or advertisers are promoting a particular product, for example, they might create dozens of apparently objective product review websites that just happen to give their own product the highest possible praise [36–38]. Manufacturers of products that compete with that product could play the same game, of course, but in each case, a true “system” of reviews has been deployed–a far more nefarious form of influence than the single blog post composed by someone expressing his or her own views.

Early recommender systems–described in the early 1990s –generally relied on two different strategies for making recommendations: “Content-based” systems recommended content based on the properties of content that a user selected in the past, whereas “collaborative-based” systems recommended content based on choices that had been made by people who were similar to the present user [39]. “Hybrid” systems used both methods [40]. By the mid 2000s, such systems were being optimized based on ever-expanding bodies of information being collected about users, specifically by making use of “user profiles that contain information about users’ tastes, preferences, and needs. The profiling information can be elicited from users explicitly, e.g., through questionnaires, or implicitly–learned from their transactional behavior over time” [41]. As marketers and leaders knew long before the internet was invented, the more you know about people, the easier it is to influence them [2,42–44]. The internet dramatically increased the rate at which information about people could be collected, and that information, in turn, has increased the power of recommender systems.

#### 1.2.1 Voting advice applications

Voting advice applications (VAAs)–also known as “online vote selectors”–are special recommender systems that use questionnaires to guide people’s votes and party affiliations. An early VAA was simply a paper-and-pencil test called the *StemWijzer*, used before elections in The Netherlands in the late 1980s [45–47]. It asked for participants’ views on various election-related issues, and based on their responses, it matched them with suitable candidates or political parties. In the late 2000s, research showed that the German *Wahl-O-Mat* questionnaire system was effective in mobilizing people to vote [48, cf. 49]. VAA methodology has been widely used across Europe to impact voters, especially over the past decade or so [46,50,51]. According to a 2009 study, 40% of voters in the 2006 national election in The Netherlands used online VAAs to guide their votes [52]. The study concluded that VAAs “had a modest effect on electoral participation and a substantial effect on party choice, especially among undecided voters” [52]. Other studies have demonstrated how various aspects of the construction of the questionnaire can impact voters differentially [53–56].

A meta-analysis of 22 VAA studies assessing data obtained from more than 70,000 users in nine countries concluded that VAAs significantly increased voter turnout, had a significant impact on voter choices, and produced modest increases in voter knowledge [57]. Again, mainly in Europe, VAAs have apparently impacted millions of voters [55], and researchers continue to study how various factors, such as the wording of questions, increase or decrease the impact of a VAA.

To our knowledge, the scientific literature on VAAs focuses exclusively on legitimate questionnaires that were designed to increase voter turnout and improve the quality of voter decisions. We have not found published experiments in which researchers used questionnaires dishonestly to try to shift votes or opinions, but we did find a blog post on Medium (not peer reviewed) in which the author reported testing the fairness of iSideWith.com by completing the website’s quiz with random answers [58]. The author concluded that the website gave biased results, but the findings were marginal, and the methodology was inadequate, in our view.

As we proceed, we will address a question that naturally comes to mind when one recognizes the power that questionnaires have to impact voters: Could VAA-type online instruments be designed to shift votes dishonestly–that is, in a way that is statistically biased toward one candidate or party? If so, could such tools impact voters in such a way that prevents them from becoming aware that they have been manipulated?

In the first part of the present study, we sought to identify websites that might be using online questionnaires dishonestly–that is, in ways that make recommendations to users that do not accurately reflect their answers to the questionnaire they completed. Do such websites exist? If so, do they violate existing laws or regulations, such as deceptive advertising or consumer fraud laws?

In the second part of the study, we describe an experiment in which an intentionally misleading VAA-type questionnaire was deployed in an attempt to shift opinions and voting preferences. Specifically, we first asked users some questions and then made recommendations while ignoring the user’s responses to the questionnaire. We sought to determine the extent to which such a procedure can shift opinions and voting preferences. We also sought to determine whether our participants were aware that they were being influenced unfairly.

## 2. Investigation 1: Examining the level of statistical bias in actual online opinion matching websites

We began our investigation by using the Brave search engine (to protect our privacy) to locate a variety of “online quizzes” (or “online questionnaires”), looking especially for quizzes of a political nature, such as quizzes that purported to match users with particular candidates or political parties. We then wrote code (in Python) that simulated a human user–in other words that clicked at human speed and that paused after it completed a quiz and submitted its answers [59–61]. Our bots took each online quiz repeatedly (generally, 300 times), and recorded the random answers our bots supplied (numerical answers to multiple-choice questions) and the recommendations the website gave. We did not attempt an exhaustive survey of such quizzes; rather, we examined only enough to yield two quizzes that gave us recommendations that were biased at a significance level under 0.001. To find these two, we had to examine a total of 15 websites. The 13 websites that appeared to give us relatively fair results are listed as S1 Text in our Supporting Information, and our Data Availability statement explains how readers can access our raw data and the Python scripts we used to access website quizzes.

### 2.1 Website 1: My political personality

#### 2.1.1 Website 1: Methods

The first of the two websites we found which appeared to give statistically biased results was https://mypoliticalpersonality.org (S2 Text), a website maintained by My Political Personality, a non-profit “voter empowerment group,” which promised to assist users in determining which of four political parties–Democrat, Republican, Libertarian, or the Green Party–was the best match for their political views. The website did so by having the user complete its “Political Personality Test,” a 15-item Likert-scale test. The website included an informal disclaimer, noting that its questionnaire was “just a fun and voluntary personality quiz–not a statement of fact.” S3 and S4 Texts show website information and its nonpartisan statement.

It matched people to a political party–just one–by revealing a user’s “political personality,” where each personality had been pre-matched (using a methodology that was not described) with a political party. See S5 Text for an example of the website’s quiz results page.

As we did for all the websites we examined, we began our investigation informally by completing the quiz manually a few times, looking for indications that the recommendations made after we completed a quiz might be biased–in this case, toward one political party. Again, we emphasize that this process was informal and exploratory only.

Because this questionnaire seemed suspect, we then customized a Python script (obtained from the Selenium WebDriver library, accessible at https://www.selenium.dev/documentation/webdriver/), to (a) clear cache and cookies, (b) reopen the tab, (c) retake the quiz by responding at random, and (d) record the results. We repeated this process 300 times. We did so in the present instance in two sessions, the first on January 4, 2022, and the second on January 15, 2022.

#### 2.1.2 Website 1: Results

Table 1 shows the frequency with which each of the political parties was recommended to the user over the course of the 300 trials. If both the questionnaire and the computation of results were completely fair, one would expect all four parties to be recommended approximately 75 times. The Republican and Libertarian parties were each recommended the expected number of times (approximately), but the Green party was never recommended, and the Democratic party was recommended roughly twice the number of expected times. The differences between the four frequencies were highly significant (*Χ*^*2*^ = 139.68, *df* = 3, *p* < 0.001), and so was the pairwise difference between the recommendations made for the Democratic and Republican parties (*z* = 5.05, *p* < 0.001).

**Table 1:** Investigation 1: Frequencies and percentages from party recommendations.

Party	Frequency	Percent	Cumulative Percent
Democrat	144	48.0	48.0
Republican	84	28.0	76.0
Libertarian	72	24.0	100.0
Green	0	0	100.0
Total	300	100.0	-

Our findings should not be interpreted as denigrating My Political Personality in any way. We neither state nor imply that our findings reflect opinions or policies of My Political Personality or its employees or affiliates, or even that the results we found in January, 2022, would still be found today. As of this writing (August 7, 2024), the website of My Political Personality has been changed to PoliticalPersonality.org, and the quiz is, as far as we can tell, no longer offered.

### 2.2 Website 2: Pew research center

#### 2.2.1 Website 2: Methods

The second quiz we found that led to apparently biased results proved to be surprising. A small, independent group calling itself “My Political Personality” posted the quiz described above; the names of the website creators were not listed. But the second suspect quiz we found was posted by the Pew Research Center, a highly respected nonprofit organization that identifies itself as a “nonpartisan fact tank” that values “independence, objectivity, accuracy, rigor, humility, transparency and innovation.” Of interest here is their “Political Typology Quiz,” still accessible at https://pewresearch.org/politics/quiz/political-typology/.

This quiz consists of 16 multiple choice questions and promises to match the user with one–just one–of nine political orientations: Progressive Left, Establishment Liberals, Democratic Mainstays, Outsider Left, Stressed Sideliners, Ambivalent Right, Populist Right, Committed Conservatives, or Faith and Flag Conservatives.

Our procedure for evaluating this quiz was identical to the procedure described for the “Political Personality” quiz we described above. The evaluation was conducted in four sessions between January 11, 2022 and January 15, 2022.

#### 2.2.2 Website 2: Results

Table 2 shows the frequencies of the political recommendations that were made. If the questionnaire had been constructed fairly, and if it had been scored fairly, we might expect it to have recommended each of the nine political orientations about 33 times. In fact, the frequencies varied from 0 (Progressive Left) to 102 (Ambivalent Right) (*Χ*^*2*^ = 219.60, *df* = 8, *p* < 0.001).

**Table 2:** Investigation 1: Frequency and percentages from typology recommendation.

Typology	Frequency	Percent	Cumulative Percent
Progressive Left	0	0.0	0.0
Establishment Liberals	52	17.3	17.3
Democratic Mainstays	16	5.3	22.7
Outsider Left	7	2.3	25.0
Stressed Sideliners	28	9.3	34.3
Ambivalent Right	102	34.0	68.3
Populist Right	35	11.7	80.0
Committed Conservatives	37	12.3	92.3
Faith and Flag Conservatives	23	7.7	100.0
Total	300	100.0	-

When we divided the nine categories into the three conventional groupings for political leaning–left, middle, and right–we again found apparently biased counts favoring conservatives (Table 3). The pairwise left/right difference was highly significant (*z* = 10.00, *p* < 0.001).

**Table 3:** Investigation 1: Frequency and percentages from categorized typology recommendation.

Group	Frequency	Percent	Cumulative Percent
Left	75	25.0	25.0
Moderate	28	9.3	34.3
Right	197	65.7	100.0
Total	300	100.0	-

Our findings should not be interpreted as denigrating Pew Research in any way. We neither state nor imply that our findings in any way reflect opinions or policies of Pew Research or its employees or affiliates, or even that the results we found in January 2022, would still be found today.

## 3. Investigation 2: A randomized, controlled OME experiment

Given the possibility that biased questionnaires (or biased scoring methods for questionnaires) might exist online, we conducted a randomized, controlled, counterbalanced, double-blind experiment that allowed us to quantify the possible impact that highly biased questionnaire scores might have on people’s opinions and voting preferences. We conjectured that scores favoring one political candidate would be able to shift voting preferences substantially while having less impact on people’s opinions about the candidate; see our Results and Discussion sections for more information about these issues.

### 3.1 Methods

#### 3.1.1 Ethics statement

The federally registered Institutional Review Board (IRB) of the sponsoring institution (American Institute for Behavioral Research and Technology) approved this study with exempt status under HHS rules because (a) the anonymity of participants was preserved and (b) the risk to participants was minimal. The IRB is registered with OHRP under number IRB00009303, and the Federalwide Assurance number for the IRB FWA00021545. Informed written consent was obtained as specified in the Procedure section of Investigation 2.

#### 3.1.2 Participants

A total of 773 demographically diverse, eligible US voters between ages 18 and 92 participated in the experiments. The sample was provided by CloudResearch, a company that draws subjects from Amazon’s Mechanical Turk subject pool, screening out bots and other suspect entities. Demographic characteristics of the sample are delineated in S1 Table. Before cleaning, our sample consisted of 816 individuals. One was removed because that person indicated that his or her English fluency was under 6 on a scale from 1 to 10 (where 1 was labeled “Not fluent” and 10 was labeled “Highly fluent”), and 42 were removed because they indicated that their level of familiarity with one or both of the two Australian political candidates mentioned in the study was greater than 3 on a scale from 1 to 10, where 1 was labeled “Not familiar at all” and 10 was labeled “Very familiar.” After cleaning, the mean familiarity level for our first candidate, Scott Morrison, was 1.15 (*SD =* 0.44), and the mean familiarity level for our second candidate, Bill Shorten, was 1.07 (0.30).

#### 3.1.3 Procedure

Using a pre-post experimental design developed by Epstein and his associates for quantifying bias in online manipulations [5–7], participants were randomly assigned (without their knowledge) to four different groups as they enrolled in the study on December 8th, 2021, December 14, 2021, or January 3, 2022. The combination of random assignment and cleaning left us with slightly uneven *n*s in each group (Table 4).

**Table 4:** Investigation 2: *n* of groups.

Group	*n*
**1.** 8 questions, high readability	197
**2.** 8 questions, low readability	197
**3.** 16 questions, high readability	187
**4.** 16 questions, low readability	192

Before beginning the experiment, all participants were given basic information about the procedure and about their rights as subjects and then asked for their informed consent to proceed (S6 Text). They were then given basic instructions about how to proceed and then shown short paragraphs about the two political candidates running for Prime Minister of Australia in 2019: Scott Morrison and Bill Shorten. The order of the names was counterbalanced throughout the study. Each paragraph was deliberately bland in tone and approximately 120 words in length (S7 Text). Then all participants were asked three opinion questions about each candidate and asked to reply on 10-point scales: One question asked how much they liked each candidate, the second asked how much they trusted each candidate, and the third asked for their overall impression of each candidate (S1 Fig shows the questions and scales).

Below those questions, participants were asked to indicate on an 11-point scale (labeled from 5 to 0 to 5) which candidate they would likely vote for it they had “to vote today.” Finally, they were asked, in a forced-choice question, to indicate which candidate they would likely vote for it they had “to vote right now.”

Participants were then asked to complete a short questionnaire that would measure their political views on a number of subjects, after which they were shown how closely their answers matched the views of the political candidates (more about this below).

Following the quiz and the scoring, all participants were asked the same eight questions they had been asked before the quiz (three opinion questions for each candidate, followed by the 11-point scale showing voting preference, followed by the forced-choice vote question).

Finally, all participants were asked whether anything about the experiment “bothered” them. If they responded “yes,” they could then type freely into a text box, expressing their concerns. This is where we ultimately looked for indications that participants showed some awareness of bias in the content they had been shown (particularly in the quiz or scores they had been shown). We could not directly ask them about whether they detected bias, because a leading question of this sort would artificially inflate the detection rate [62].

Participants were then thanked for their participation, given a code they could use to receive their payment, and given an email address they could use to withdraw their data from the experiment or to address questions to the researchers.

**Fig 1:**
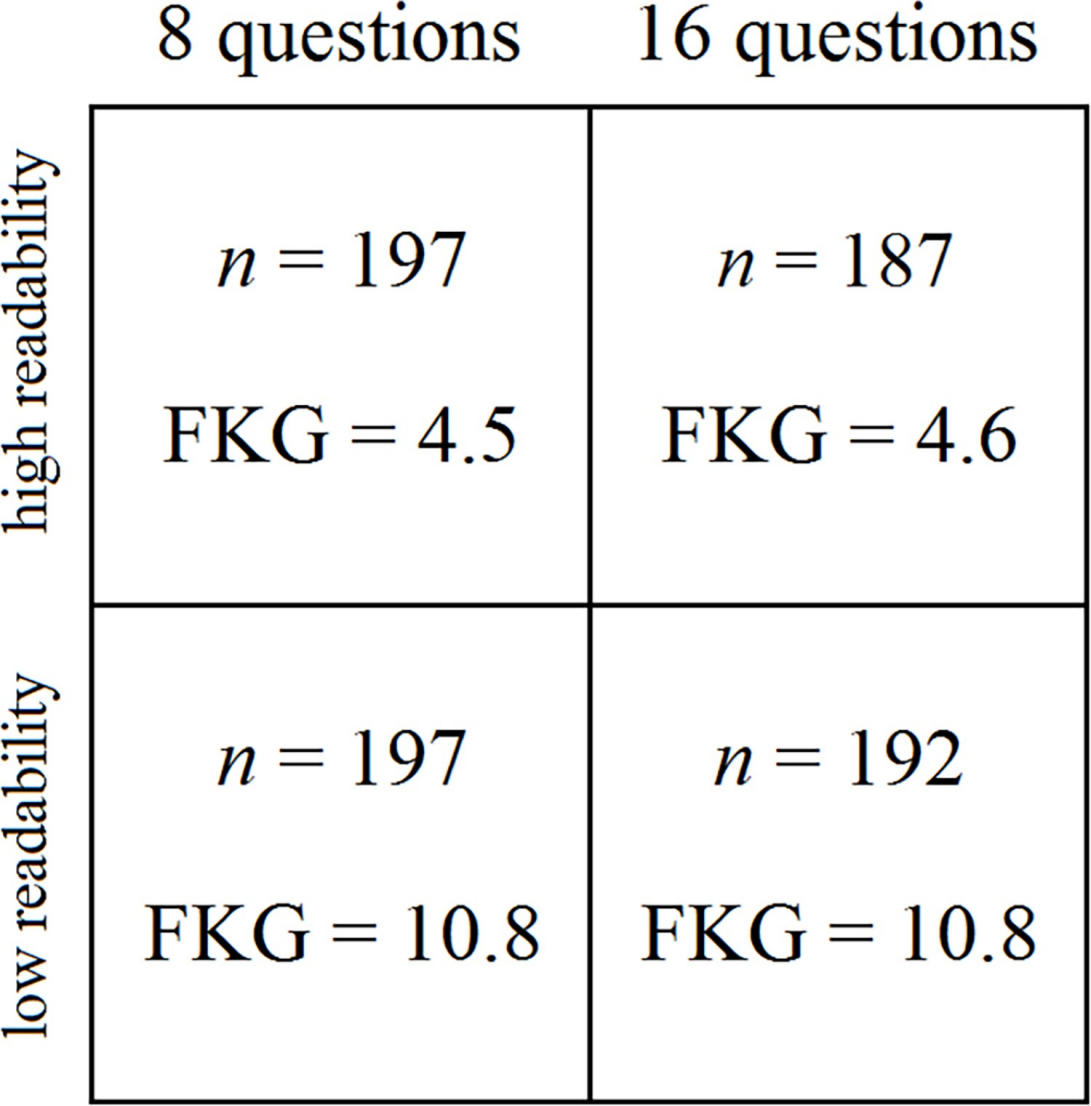
2-by-2 factorial design showing two levels of quiz readability (low and high) and two quiz lengths (8 questions and 16 questions). The *n* for each of the four groups is shown in each box, along with the Flesch-Kincaid Grade Level (FKG) of the content.

*The four groups*. Fig 1 depicts the 2-by-2 factorial design employed in the study. Because recent studies, particularly in the EU, have found that the impact of election-related quizzes varies with the structure and content of such quizzes [53–56], we elected to vary both the content and length of our quiz. Participants were given either high- or low-readability quizzes, and the quizzes were either 8 or 16 questions in length (Fig 1). S8 to S11 Texts show the different quiz questions for each of the four groups. S2 Fig shows the quiz homepage, and S3 Fig shows an example of the website page during the quiz-taking process.

At first glance, our design might be viewed as having a 3-by-2-by-2 factorial structure, because all participants were also randomly assigned to three different candidate bias groups: pro-Candidate-1 (Morrison), pro-Candidate 2 (Shorten), or control (neutral, favoring neither candidate). However, as we will explain below, our analysis of the data combined the two candidate bias groups into one group, and it was only for that group that the 2-by-2 factorial design applied, so it would be misleading to characterize our experimental design as having three separate dimensions.

Following the quiz, participants were shown an animated loading bar for 5 sec to give the impression that their responses were being processed (S4 Fig). Then, participants who had been assigned to the pro-Candidate-1 group (Morrison) were informed that 85% of their answers matched Morrison’s views and that 25% of their answers matched Shorten’s views (S5 Fig); participants who had been assigned to the pro-Candidate-2 group (Shorten) were informed that 85% of their answers matched Shorten’s views and that 25% of their answers matched Morrison’s views; and participants in the control group were informed that 42% of their answers matched the views of each candidate (S6 Fig). Note that the 85% and 25% values sum to a value over 100% because, presumably, there was some overlap in agreement between the two candidates. That said, all three of the percentages we used in this experiment were chosen somewhat arbitrarily.

### 3.2 Results

The main measure of interest in experiments that use a pre-post manipulation design to measure changes in voting preferences is vote manipulation power, or VMP–the post-manipulation percentage increase in the number of people choosing to vote for the candidate favored in their group [5]. This is calculated by combining the data in the two bias groups. For details about how to compute VMP, see S12 Text.

In the present experiment, the overall VMP for the four quiz groups combined was 75.5% (95% CI, 70.3–80.7%; McNemar’s test, *p* < 0.001), which is high compared with VMPs found in comparable experiments on new forms of manipulation made possible by the internet [5–9]. S2 to S5 Tables show VMPs broken down by educational attainment, gender, age, and race/ethnicity. VMPs in the four quiz groups ranged from 50.7% to 95.2% (Table 5), the latter value being the highest value our research group has ever found in comparable experiments on online influence. The percentage of users who appeared to perceive some degree of bias in the content they were shown was also notable in this experiment: not a single participant claimed to observe any bias in the content.

**Table 5:** Investigation 2: VMP for each of the four groups.

Group	Total *n*	Bias Groups *n*	VMP (%)	95% VMP Confidence Interval	McNemar’s Test *X*^*2*^	*p*
**1.** 8 questions, high readability	197	129	77.3	67.2–87.4	45.455	< 0.001
**2.** 8 questions, low readability	197	132	95.2	90.0–100.0	58.017	< 0.001
**3.** 16 questions, high readability	187	121	50.7	39.1–62.3	29.167	< 0.001
**4.** 16 questions, low readability	192	128	82.0	72.3–91.6	44.463	< 0.001
**Total**	773	510	75.5	70.3–80.7	182.066	< 0.001

**Fig 2:**
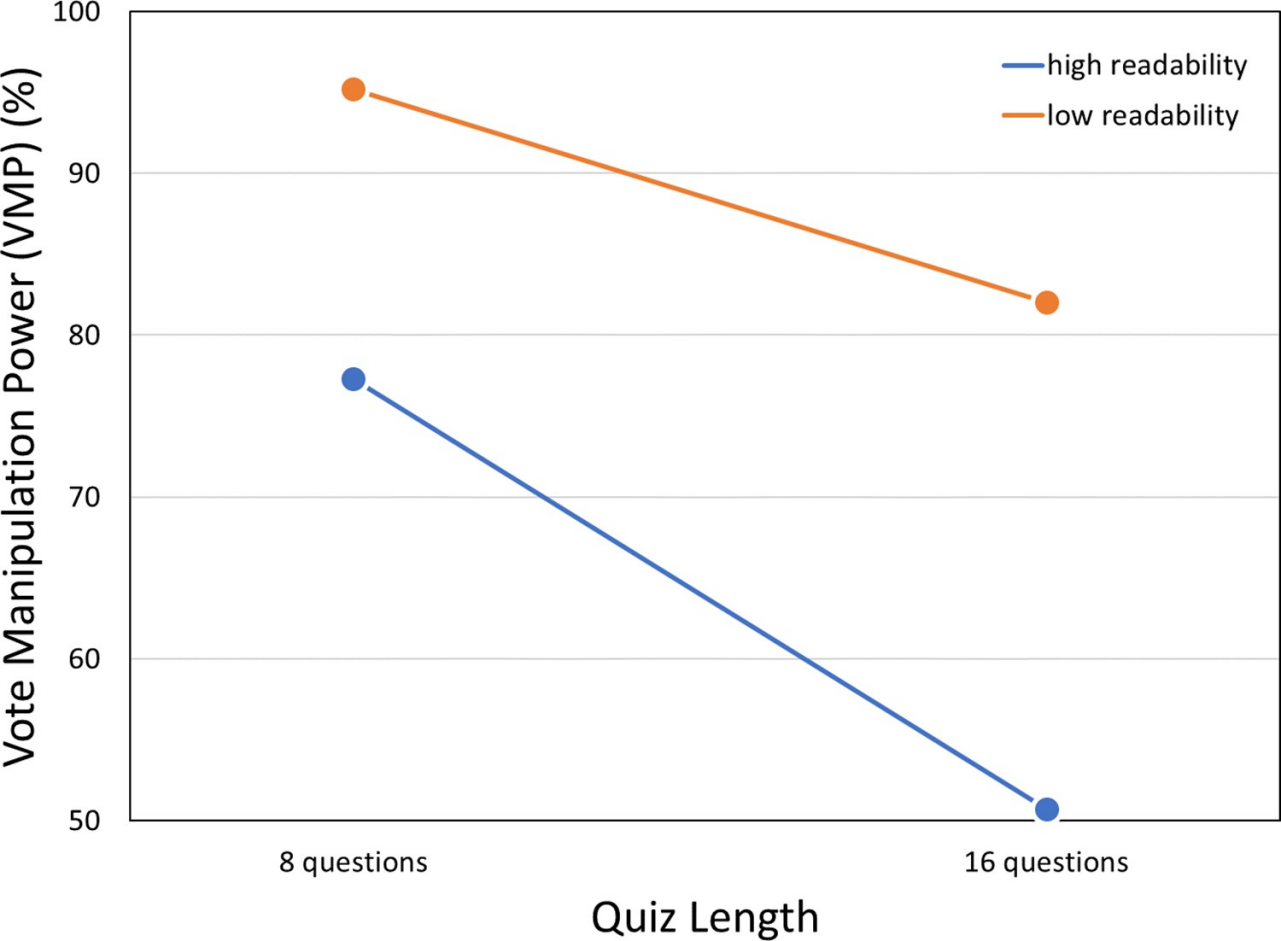
VMPs broken down by quiz characteristics. Pre-post shifts in voting preferences as expressed by VMPs suggest that low-readability quizzes produce greater shifts toward the favored candidate than high-readability quizzes do, and that shorter quizzes produce greater shifts than longer quizzes do. See text for details.

Regarding the possible differential impact of the quiz characteristics, the VMPs for the four subgroups in the 2-by-2 factorial design (Table 5 and Fig 2) suggest main effects for both the quiz length (with the shorter quiz having the greater impact) and readability (with low readability having the greater impact), with little or no interaction between these effects. To our knowledge, a standard ANOVA cannot be performed on our VMPs because the same VMP applies to all members of a group (see S12 Text for the calculation method). VMPs are percentages, not means, so no simple measure of individual variability underlies them. We can estimate the magnitude and significance of main effects, however, with *z*-tests, as shown in Table 6. Both effects were highly significant, with readability the larger effect.

**Table 6:** Investigation 2: Comparison of VMPs for two levels of quiz length and two levels of readability.

Factor	Treatment	*n*	VMP (%)	*z*	*p*
Quiz Length	8 questions	261	86.0	5.213	< 0.001
	16 questions	249	65.2	-	-
Readability	low	260	88.7	6.347	< 0.001
	high	250	63.5	-	-

**Fig 3:**
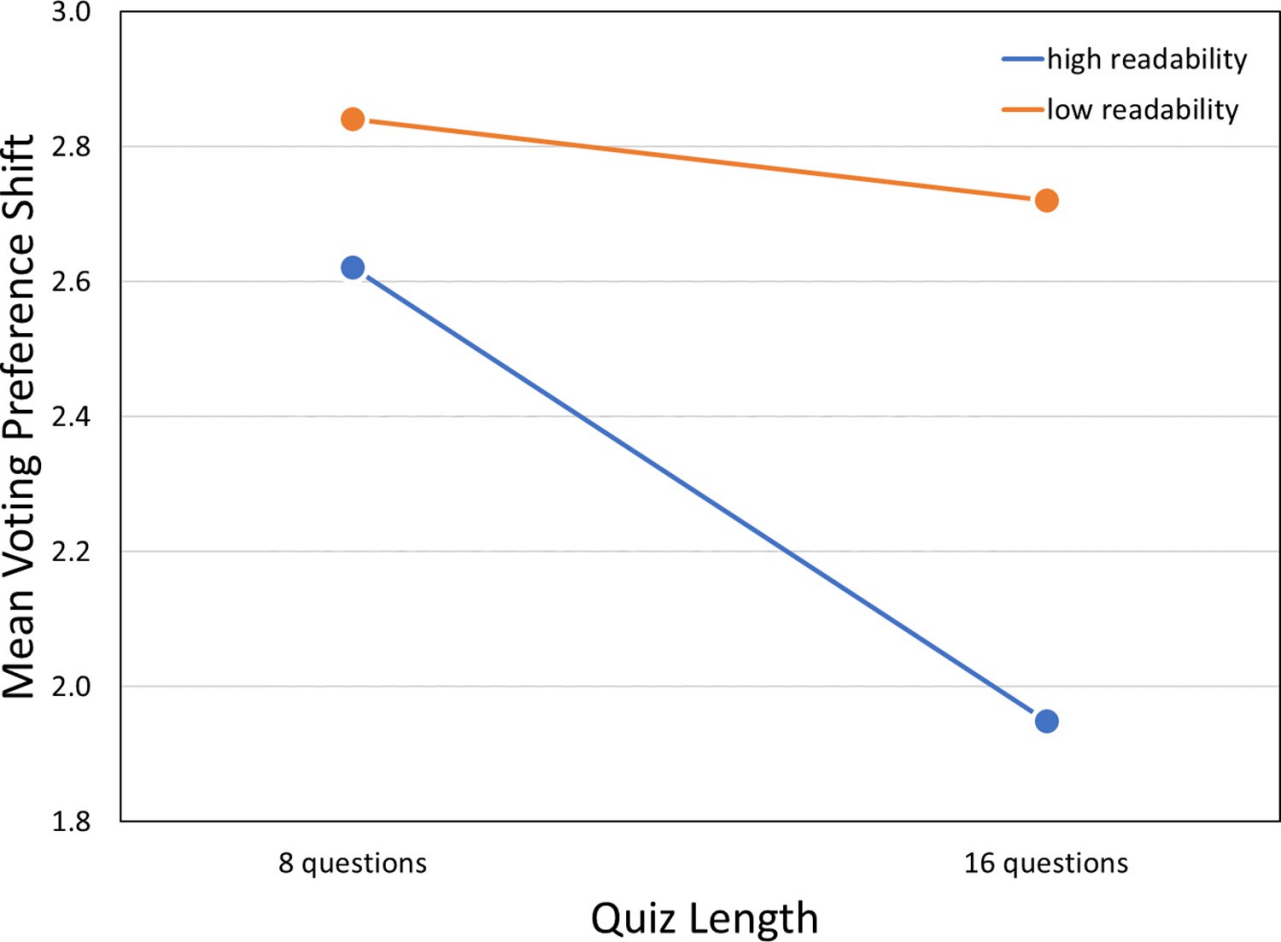
Voting preference shifts on the 11-point scale broken down by quiz characteristics. Pre-post shifts in voting preferences as expressed on an 11-point scale suggest that low-readability quizzes produce greater shifts toward the favored candidate than high-readability quizzes do, and that shorter quizzes produce greater shifts than longer quizzes do. See text for details.

Voting shifts on the 11-point scale (for the two bias groups combined) were also relatively large and occurred in the predicted direction (Table 7). Main effects for quiz characteristics were marginal, with no evidence of interaction (Table 8 and Fig 3). We found no pre-post differences in voting preferences as expressed on the 11-point scale for participants in the neutral groups (S6 Table).

**Table 7:** Investigation 2: Pre- and post-manipulation votes on 11-point scale (5 to 0 to 5) by quiz group*.

	Pre-manipulation		Post-manipulation					
Quiz Group	Bias Groups; Mean (*SD*)	Neutral Group; Mean (*SD*)	Bias Groups; Mean (*SD*)	Neutral Group; Mean (*SD*)	Bias Groups; Mean Shift	Neutral Group; Mean Shift	Cohen’s *d*^†^	Mann-Whitney *U*^‡^
**1.** 8 questions, high readability	0.09 (2.64)	0.47 (2.70)	2.71 (2.30)	0.69 (2.14)	2.62	0.22	1.06	1,801.50***
**2.** 8 questions, low readability	0.02 (2.63)	0.42 (2.49)	2.86 (1.94)	0.40 (2.28)	2.84	-0.02	1.23	1,297.50***
**3.** 16 questions, high readability	0.54 (2.45)	0.02 (2.59)	2.49 (2.13)	-0.09 (2.57)	1.95	-0.11	0.85	1,483.50***
**4.** 16 questions, low readability	-0.22 (2.43)	-0.16 (2.85)	2.50 (2.54)	0.05 (2.80)	2.72	0.21	1.09	1,415.50***
**Total**	0.10 (2.55)	0.19 (2.66)	2.64 (2.24)	0.27 (2.45)	2.54	0.08	1.06	23,959.50***

^*^Positive means indicate shifts in the direction of the favored candidate after correcting for counterbalancing.

^**†**^Cohen’s *d* effect sizes were calculated using the means and standard deviations of the pre- and post-manipulation opinion ratings for the bias groups combined.

^‡^Mann-Whitney *U* tests were conducted for the score shifts between each bias group and each neutral group.

*** *p* < 0.001

**Table 8:** Investigation 2: ANOVA of vote preference score shifts on the 11-point scale for two factors: Quiz length and readability.

Effect	Sum of Squares	*df*	*F*	*p*
Quiz Length	19.045	1	2.858	0.092 NS
Readability	29.871	1	4.482	< 0.05
Quiz Length × Readability	9.814	1	1.473	0.226 NS

We also found significant pre-post differences in opinions people expressed about the favored candidate (the candidate we identified as a great match to their quiz answers), although effect sizes were relatively low (Table 9). In an ANOVA, we found no main effects or interactions reflecting differential characteristics of the quizzes (S7 Table).

**Table 9:** Investigation 2: Pre- and post-manipulation opinion ratings of favored candidates (for the bias groups combined).

Group	Opinion	Pre	Post	Diff	Cohen’s *d*^†^	*z* ^‡^
**1.** 8 questions, high readability	Impression	7.28 (1.85)	7.88 (1.77)	0.60	0.33	-4.597***
	Trust	6.44 (1.92)	7.09 (2.03)	0.65	0.33	-5.068***
	Likeability	7.05 (1.89)	7.53 (1.94)	0.48	0.25	-3.446**
**2.** 8 questions, low readability	Impression	7.33 (1.66)	7.98 (1.45)	0.65	0.42	-4.784***
	Trust	6.31 (1.93)	6.95 (1.97)	0.64	0.33	-5.488***
	Likeability	7.18 (1.66)	7.69 (1.50)	0.51	0.32	-4.295***
**3.** 16 questions, high readability	Impression	7.02 (1.82)	7.52 (1.79)	0.50	0.28	-4.452***
	Trust	6.31 (2.20)	6.82 (2.19)	0.51	0.23	-3.792***
	Likeability	7.07 (1.91)	7.23 (1.97)	0.16	0.08	-1.387
**4.** 16 questions, low readability	Impression	7.13 (1.89)	7.68 (1.70)	0.55	0.30	-3.996***
	Trust	6.18 (1.96)	6.91 (1.90)	0.73	0.38	-5.861***
	Likeability	7.08 (1.87)	7.57 (1.63)	0.49	0.28	-4.071***
**Total**	Impression	7.20 (1.75)	7.83 (1.64)	0.63	0.33	-8.873***
	Trust	6.48 (1.96)	7.03 (1.91)	0.55	0.32	-10.113***
	Likeability	7.24 (1.80)	7.67 (1.73)	0.43	0.23	-6.629***

^†^Cohen’s *d* effect sizes were calculated using the means and standard deviations of the pre- and post-manipulation opinion ratings for the favored candidate.

^‡^z values represent Wilcoxon signed ranks test comparing pre- to post-manipulation opinion ratings for the favored candidate.

*** *p* < 0.001, ** *p* < 0.01.

Of special note, no participants in either of the bias groups reported any awareness of bias in the content they viewed in this study, or in the scores they were shown after they completed the quiz.

## 4. Discussion

In our view, this study produced two quite remarkable results. First, it produced the largest shifts in voting preferences (as measured by VMP, which is calculated from answers to a forced-choice question: “If you had to vote right now, who would you vote for?”) we have ever observed after having conducted more than 10 years of studies of this sort [5–9]–shifts between 50.7% and 95.2%. Second, it is the only online manipulation study we have ever conducted (without the use of masking procedures) that apparently produced no awareness of bias by participants. Why would a quiz-based manipulation produce such a large impact with so little cost?

We think the answer is fairly obvious. A quiz posted with the apparent purpose of helping someone make an informed decision provides a service, at least from the point of view of most, if not all, users, which is why marketers use quizzes for lead generation, branding, data gathering, and other purposes [63–66]. While completing a quiz, the user is not necessarily exposed to biased content and has no factual basis for suspecting that a manipulation is about to occur. The manipulation occurs only *after* the quiz is completed, when the user is presented with inaccurate information about his or her scores. At that point, the user has no way to evaluate the accuracy of the score. Bear in mind that people who take quizzes to help them make decisions (about political candidates, guitars, vacation spots, or just about anything else) almost certainly lack the knowledge they need to make an informed decision; that, presumably, is why they are taking the quiz. An online quiz is, in effect, an ideal tool both for attracting users who are vulnerable to manipulation and for causing an invisible manipulation to occur.

Because OME can apparently produce large shifts in opinions and voting preferences without user awareness, we wonder why–at least as far as we can tell–researchers have consistently evaluated the impact of quizzes based on users’ actual answers and scores. Because it is such a simple matter to ignore those answers and fake those scores, and because the internet is awash with quizzes of all sorts, why have researchers not addressed this issue? This raises the question we asked in our first investigation (above): Are people–or organizations, companies, or political parties–indeed manipulating users by showing them biased results that are largely or entirely independent of their answers? We presented two examples of online quizzes that appear to be showing users statistically biased results. One quiz–“My Political Personality”–was posted by a small, independent organization of the same name, and the other quiz was posted by the venerable Pew Research Center. It is possible that neither group was aware of the bias in its quiz–that the bias was entirely accidental and unintentional. Even if we give both organizations the benefit of the doubt, however, *our findings suggest that these quizzes are still shifting opinions systematically*.

The specific type of bias we are referring to here is *algorithmic bias*, which results in output that creates an unfair, systematic advantage for certain groups, whether deliberate on the programmer’s part or not [67]. Other types of bias that might be present in online opinion matching questionnaires include “question-wording bias” [68], in which question phrasing might favor certain candidates, ideologies, or products over others [69, cf. 70], and “response option bias,” in which the available response options might favor certain candidates, ideologies, or products over others [71, cf. 69,72,73]. Recommender systems that produce biased results are a cause for concern because they can lead to dampened competition in the marketing and political arenas [74,75].

Our findings also demonstrate how easily online quizzes could be used to shift opinions and votes on a massive scale. We have no evidence that online quizzes are being used that way, but it is notable here that in the months preceding national elections in the US in 2016 and 2020, a number of election-related quizzes were posted on high-traffic websites such as https://WaPo.com and https://BuzzFeed.com. Even Tinder, known mainly as a “hookup” website where people swipe left or right to indicate whether they are attracted to someone, deployed a “Swipe the Vote” feature in March, 2016, to help its 50 million users decide whom to vote for in November [76] (S7 Fig). Notably, according to https://OpenSecrets.org, in 2016, 89.3% of the political donations from Tinder’s parent company at that time (InterActiveCorp) went to just one of the two major political parties in the US [77]. Our data suggest that if Tinder had been using its Swipe-the-Vote feature dishonestly, it could have shifted–at least temporarily–the voting preferences of between 1.3 and 2.4 million undecided voters (see S13 Text for how we arrived at this estimate).

### 4.1 Limitations and future research

This brings us to two likely limitations of OME. First, we have no evidence that this effect leaves a lasting impact on a user’s voting preferences. The impact will vary according to how vulnerable someone is to this type of subtle persuasion. It might have a lasting impact on only a small proportion of voters–a matter to be explored in future research. The voter most likely to be influenced by a biased quiz site is the one who, in a last-minute attempt to get off the fence, takes the quiz on Election Day or perhaps the day before. If so, that would greatly limit the impact of the quiz.

Second, OME–to the extent that it is being used at all–is probably being used competitively. Platforms capable of employing new forms of influence such as the search engine manipulation effect (SEME) [5], the search suggestion effect (SSE) [8], and the targeted messaging effect (TME) [7] can expose people to similarly biased content hundreds of times before an election as people conduct search after search, or as they scroll, over and over again, through Twitter feeds (or “X” feeds, if you prefer). People are unlikely, however, to complete similarly biased questionnaires repeatedly in the months leading up to an election. Unlike SEME, SSE, and the video manipulation effect (VME) [9], OME is an inherently competitive manipulation. It is not controlled exclusively by a tech monopoly; biased quizzes can be posted by just about anyone. In that sense, biased quizzes are more like blogs than they are like search results. That said, if any of the Big Tech platforms started using online quizzes to shift opinions or votes, or began promoting certain quizzes while suppressing others, they could conceivably shift millions of votes with no one able to counteract their actions.

Is it legal to shift opinions, purchases, or votes by giving people fake scores on quizzes? We have not been able to find any relevant cases in the US, but if OME becomes a popular tool for shifting large numbers of votes in elections, it is conceivable that political parties or public officials might start searching the law books for relevant laws and regulations. Quizzes used to manipulate people online might violate provisions of the Federal Election Commission Act, the Federal Trade Commission Act, the Consumer Protection Act, or the Uniform Deceptive Trade Practices Act, as well as any number of state laws or regulations. This is a matter for legal scholars to research, not social scientists.

One also might wonder about the quiz itself. Given that our quiz contains no information about the candidates, why should different quiz characteristics have *any* differential impact on the outcome of our manipulation? Apparently, a shorter quiz, or a quiz that is more difficult to read, makes people somewhat more vulnerable to the manipulation (seeing fake scores that favor one candidate or another). One might speculate that people will consider a verbose quiz to be more substantive, but shouldn’t they also take a *longer* quiz more seriously? Our design did not allow us to explore such issues, but they might be worth exploring in future studies. Generally speaking, longer test instruments have been shown to produce less consistent or honest responses [78], and, surprisingly, more verbose test instruments have been shown to produce responses that are *more* consistent and honest [79]. Both findings are consistent with our new findings, but we still find it surprising that different characteristics of our quizzes had any differential effects at all–another matter to be explored in future research.

- One might also be concerned about the method we used to evaluate the fairness of online tests– 15 quizzes in all (see Investigation 1). Will random responding necessarily tell you that a quiz is statistically biased? We argued that random responding should produce scores that don’t favor any particular political party or candidate (or guitar brand, for that matter). We acknowledge, however, that a questionnaire might be constructed in good faith and without conscious bias that will not survive the random-responding test. That said, recall that in our evaluation of the Pew quiz, we were *never* labeled Progressive Left–one of nine possible labels we might have received–even though we completed the quiz 300 times. If we make the reasonable assumption that by responding at random, we should be labeled Progressive Left 1/9th of the time, then the probability that we are *never* labeled that way after 300 trials is a disturbing 4.5 × 10^−16^. Of course, the quiz might have been legitimately constructed so that that label is rarely applied, perhaps because Progressive Leftists are near the tail end of a normal distribution. But even assuming that random responding should produce that label only 1/50th of the time, the probability that we are *never* labeled that way is still only 0.002.

An exploration of how online questionnaires should be constructed is beyond the scope of this paper, and it is also irrelevant to the central point we are raising, namely that questionnaires can be posted online that can easily shift people’s opinions and voting preferences without their knowledge. Because the experience of completing an online questionnaire is normally ephemeral, with no record being kept of the experience, this type of manipulation, like SEME and other online effects we have studied, leaves no paper trail for authorities to trace. We cannot go back in time to measure the possible impact of Tinder’s Swipe-the-Vote feature. It might have had very little impact on the 2016 election (especially if it had been fair and honest in its scoring), or it might have had a significant impact; unless a whistleblower comes forward or old records are revealed, we will never know. This is why our research team has, since 2016, been building increasingly larger and more capable monitoring systems that preserve online ephemeral experiences [80–83]. In 2016, we were able to preserve and later analyze about 13,000 politically-related searches on the Google, Bing, and Yahoo search engines [80]. As of this writing (August 7, 2024), we have in recent months preserved more than 96 million ephemeral experiences on multiple platforms, and we are continuing to monitor online content 24 hours a day through the computers of a politically-balanced group of more than 15,000 registered voters in all 50 US states [84].

We also acknowledge that the magnitude of the effect we found in our experiment (Investigation 2) might be due in part to the fact that our participants were what some political scientists call “low-information” undecided voters. This is so because we used participants from the US to make judgments about political candidates from Australia. High-information undecided voters differ from low-information undecided voters in some respects [85], although, to our knowledge, there is currently no evidence that low-information undecided voters are more vulnerable to online manipulations such as OME. We do know from SEME studies, however, that low-information undecided voters are generally more vulnerable to manipulation in the search engine environment than high-information undecided voters are [5]. Again, this is an issue that can only be settled by further research.

We remind the reader that we did not in this investigation attempt to survey the internet to try to estimate the number or proportion of websites that might currently be using quizzes unfairly to manipulate people’s opinions. Rather we used a simple proof-of-concept procedure: We counted the number of websites that used quizzes that we needed to investigate in order to find just two websites that appeared to produce statistically biased results. We found those two websites among the first 15 that we examined (13.3%). We have no evidence that this same proportion of quiz-based websites is suspect throughout the internet.

Finally, we hope this study will serve as a reminder to scientists, public policy makers, and interested members of the general public that the internet is very much out of control. The content of print media has been constrained in various ways since not long after the printing press was invented, but there are still virtually no constraints on the kind of content that can be posted online. This means, among other things, that new means of manipulation that the internet has made possible can be used, and almost certainly *are* being used, to impact the thinking and behavior of billions of people in potentially destructive or self-destructive ways without their knowledge or consent [5–9]. OME matters because it is a powerful tool for shifting people’s opinions and voting preferences which appears to be completely invisible to users. If we can discover this, so can bad actors. When, in the 1990s, the internet was little more than an efficient means of digital communication between universities, it presented no great threat to humanity. Unfortunately, we allowed the internet to mushroom into a pervasive tool of surveillance, censorship, and manipulation without implementing laws and regulations to protect users, and that is where we stand today.

## Supporting information

S1 Text Investigation 1: List of relatively fair opinion matching website quizzes. (DOCX)

S2 Text MyPoliticalPersonality home page. (DOCX)

S3 Text MyPoliticalPersonality website information. (DOCX)

S4 Text MyPoliticalPersonality nonpartisan statement. (DOCX)

S5 Text MyPoliticalPersonality results page: “Social Guardian” (democratic) recommendation. (DOCX)

S6 Text Investigation 2: Informed consent. (DOCX)

S7 Text Investigation 2: Candidate biographies. (DOCX)

S8 Text Group 1: 8 questions, high readability (FKG = 4.5). (DOCX)

S9 Text Group 2: 8 questions, low readability (FKG = 10.8). (DOCX)

S10 Text Group 3: 16 questions, high readability (FKG = 4.6). (DOCX)

S11 Text Group 4: 16 questions, low readability (FKG = 10.8). (DOCX)

S12 Text Vote manipulation power (VMP) calculation. (DOCX)

S13 Text Tinder’s Swipe-the-Vote shift estimate calculation. (DOCX)

S1 Fig Investigation 2: Pre- and post-test opinion and voting questions. (DOCX)

S2 Fig DoodleMatch home page. (DOCX)

S3 Fig Investigation 2: 8-question, high readability quiz. (DOCX)

S4 Fig Investigation 2: Quiz result calculation bar. (DOCX)

S5 Fig DoodleMatch results page: Scott Morrison recommendation. (DOCX)

S6 Fig DoodleMatch results page: Neutral group recommendation. (DOCX)

S7 Fig Tinder’s “Swipe the Vote” feature home page from March 23^rd^, 2016. (DOCX)

S1 Table Demographic characteristics in Investigation 2 by quiz group. (DOCX)

S2 Table Investigation 2: Demographic analysis by educational attainment. (DOCX)

S3 Table Investigation 2: Demographic analysis by gender. (DOCX)

S4 Table Investigation 2: Demographic analysis by age. (DOCX)

S5 Table Investigation 2: Demographic analysis by race/ethnicity. (DOCX)

S6 Table Investigation 2: Pre- and post-quiz mean voting preferences on 11-point scale for neutral groups by quiz group. (DOCX)

S7 Table Investigation 2: ANOVA of opinion shifts (in the bias groups combined) for two factors: Quiz length and readability. (DOCX)
