# Supplementary material for: The “opinion matching effect” (OME): A subtle but powerful new form of influence that is apparently being used on the internet
Source: PLoS One. 2024 Sep 12;19(9):e0309897. doi: 10.1371/journal.pone.0309897 (PMC11392280; doi:10.1371/journal.pone.0309897)
Supplement: S1 Text — (DOCX) [file pone.0309897.s001.docx]

**S1 Text. Investigation 1: List of relatively fair opinion matching website quizzes**

1. <https://www.brainfall.com/which-guitar-are-you/>
2. <https://www.buzzfeed.com/shookethbb/what-fast-food-restaurant-are-you>
3. <https://www.gotoquiz.com/what_are_the_best_basketball_shoes_for_you>
4. <https://www.gotoquiz.com/which_canadian_political_party_should_you_vot>
5. <https://www.gotoquiz.com/which_guitar_brand_are_you_1>
6. <https://www.isidewith.com/>
7. <https://www.laliga.com/en-GB/news/which-laliga-santander-team-should-you-support>
8. <https://www.nflteampicker.nfl.com/>
9. <https://www.opencampaign.com/quiz>
10. <https://www.playbuzz.com/danielr51/which-nba-team-should-you-root-for>
11. <https://www.theadvocates.org/quiz/>
12. <https://www.thequiz.com/take-this-60-second-quiz-and-well-tell-you-which-smartphone-you-should-buy/>
13. <https://www.votecompass.cbc.ca/canada>
