# Supplementary material for: The “opinion matching effect” (OME): A subtle but powerful new form of influence that is apparently being used on the internet
Source: PLoS One. 2024 Sep 12;19(9):e0309897. doi: 10.1371/journal.pone.0309897 (PMC11392280; doi:10.1371/journal.pone.0309897)
Supplement: S2 Text — (DOCX) [file pone.0309897.s002.docx]

**S2 Text. MyPoliticalPersonality home page.**

<https://aibrt.org/downloads/Supp_Fig-OME-MyPoliticalPersonality_home_page.png>
