# Supplementary material for: The “opinion matching effect” (OME): A subtle but powerful new form of influence that is apparently being used on the internet
Source: PLoS One. 2024 Sep 12;19(9):e0309897. doi: 10.1371/journal.pone.0309897 (PMC11392280; doi:10.1371/journal.pone.0309897)
Supplement: S6 Text — (DOCX) [file pone.0309897.s006.docx]

**S6 Text. Investigation 2: Informed consent.**

Participant Instructions:

Thank you for your interest in our study, which is being conducted by a nonprofit, nonpartisan organization called HFE Research. We are interested in how internet research might affect the way people view politics. Here is how the study works:

First, we will ask you some basic questions about yourself. Your answers will be kept strictly confidential and are being used for research purposes only, so please be honest. To protect your privacy, we will *not* ask you for your last name.

Then we will give you some basic information about Australia's candidates for Prime Minister. Then we'll ask you a few questions about your views on the candidates.

After you have answered these questions, we'll give you the opportunity to take a quiz that will match up your views on issues with a candidate.

The entire process typically takes between 10 and 15 minutes, and most people find it to be quite interesting.

This study has been reviewed and approved by our organization's Institutional Review Board. We do not believe that your participation in this study is risky in any way, but if you encounter any problems or have any concerns, we encourage you to email the researchers at info@HFEResearch.org. After you have completed the survey you will have the option to contact us if for any reason you wish to have your data removed from the study.

PLEASE NOTE: It is important that you participate fully and honestly in every part of the study. That is the only way the study can produce meaningful results. So please don't skip anything!

To participate in this study you must check the box below to give your consent to the following:

I am 18 years or older and I understand that my participation is voluntary, that I am free to withdraw at any time, that I am providing information anonymously and that demographic information collected is confidential and cannot be used to identify me. I agree to allow the data collected to be used for future research projects, and I understand that completion and submission of this survey implies my consent to participate in the present study:

I ( ) *Do* ( ) *Do Not* give my consent and agree to the above statement.
