# Supplementary material for: The “opinion matching effect” (OME): A subtle but powerful new form of influence that is apparently being used on the internet
Source: PLoS One. 2024 Sep 12;19(9):e0309897. doi: 10.1371/journal.pone.0309897 (PMC11392280; doi:10.1371/journal.pone.0309897)
Supplement: S8 Text — (DOCX) [file pone.0309897.s008.docx]

**S8 Text. Group 1: 8 questions, high readability (FKG = 4.5).**

1. Should weed be made legal?
2. Should military spending be raised?
3. Should the COVID vaccine be mandatory?
4. Is global warming real?
5. Should taxes be raised on the super-rich?
6. Should same-sex marriage be banned?
7. Should abortion be banned?
8. Should there be stronger gun control laws?
