# Supplementary material for: The “opinion matching effect” (OME): A subtle but powerful new form of influence that is apparently being used on the internet
Source: PLoS One. 2024 Sep 12;19(9):e0309897. doi: 10.1371/journal.pone.0309897 (PMC11392280; doi:10.1371/journal.pone.0309897)
Supplement: S9 Text — (DOCX) [file pone.0309897.s009.docx]

**S9 Text. Group 2: 8 questions, low readability (FKG = 10.8).**

1. Should recreational marijuana be prohibited for everyone in the country?
2. Should government spending on the military be substantially increased?
3. Should everyone in the country be required to get the COVID-19 vaccine?
4. Should the government prioritize global climate change issues?
5. Should the government raise taxes substantially on wealthy individuals and companies?
6. Should homosexual marriage be made legal everywhere in the country?
7. Should voluntary abortion be prohibited under all circumstances?
8. Should there be more and harsher gun control laws?
