# Supplementary material for: The “opinion matching effect” (OME): A subtle but powerful new form of influence that is apparently being used on the internet
Source: PLoS One. 2024 Sep 12;19(9):e0309897. doi: 10.1371/journal.pone.0309897 (PMC11392280; doi:10.1371/journal.pone.0309897)
Supplement: S10 Text — (DOCX) [file pone.0309897.s010.docx]

**S10 Text. Group 3: 16 questions, high readability (FKG = 4.6).**

1. Should weed be made legal?
2. Should military spending be raised?
3. Should the COVID vaccine be required?
4. Is global warming real?
5. Should taxes be raised on the super-rich?
6. Should same-sex marriage be banned?
7. Should abortion be made illegal?
8. Should there be more gun control laws?
9. Should the minimum wage be increased?
10. Should there be more laws to fight racism?
11. Should bilingual education be required?
12. Should porn be banned?
13. Should the death penalty be abolished?
14. Should immigration be restricted?
15. Should nuclear weapons be banned?
16. Should the government stop trading with China?
