# Supplementary material for: The “opinion matching effect” (OME): A subtle but powerful new form of influence that is apparently being used on the internet
Source: PLoS One. 2024 Sep 12;19(9):e0309897. doi: 10.1371/journal.pone.0309897 (PMC11392280; doi:10.1371/journal.pone.0309897)
Supplement: S11 Text — (DOCX) [file pone.0309897.s011.docx]

**S11 Text. Group 4: 16 questions, low readability (FKG = 10.8).**

1. Should recreational marijuana be legalized everywhere in the country for everyone 18 and over?
2. Should government spending on the military be substantially increased?
3. Should everyone in the country be required to get the COVID-19 vaccine?
4. Should the government prioritize global climate change and global warming issues?
5. Should the government increase taxes substantially on extremely wealthy individuals and companies?
6. Should homosexual marriage be made legal for everyone in the country?
7. Should voluntary abortion be prohibited under all circumstances?
8. Should there be more and harsher gun control regulations and restrictions?
9. Should the government substantially increase the minimum wage for all workers?
10. Should the government make more rules and regulations to combat racism in the country?
11. Should bilingual education be obligatory at all public schools in the country?
12. Should the production and transmission of pornography be made illegal?
13. Should capital punishment, which is also known as the death penalty, be made illegal?
14. Should all types of immigration be made completely illegal to protect domestic jobs and security?
15. Should the manufacture and possession of nuclear weapons be prohibited?
16. Should the government reduce the volume of trade it does with mainland China?
