# Supplementary material for: The “opinion matching effect” (OME): A subtle but powerful new form of influence that is apparently being used on the internet
Source: PLoS One. 2024 Sep 12;19(9):e0309897. doi: 10.1371/journal.pone.0309897 (PMC11392280; doi:10.1371/journal.pone.0309897)
Supplement: S13 Text — (DOCX) [file pone.0309897.s013.docx]

**S13 Text. Tinder’s Swipe-the-Vote shift estimate calculation.**

We base this estimate on the following modest assumptions: (a) that several months before the Presidential election, 20% of Tinder users were undecided voters (0.2 × 50,000,000 = 10,000,000), (b) that 50% of those undecided voters tried out Tinder’s Swipe-the-Vote application (0.5 × 10,000,000 = 5,000,000), (c) that before the manipulation, if those voters had been asked a forced-choice question about how they planned to vote, they would likely have split 50/50 (2,500,000 for each candidate), and (d) that after the manipulation, between 50.7% and 95.2% of the voters in one of those groups might have shifted their preference toward the other candidate (0.507 × 2,500,000 = 1,267,500; 0.952 × 2,500,000 = 2,380,000).
