# Supplementary figures and images for: The “opinion matching effect” (OME): A subtle but powerful new form of influence that is apparently being used on the internet
Source: PLoS One. 2024 Sep 12;19(9):e0309897. doi: 10.1371/journal.pone.0309897 (PMC11392280; doi:10.1371/journal.pone.0309897)

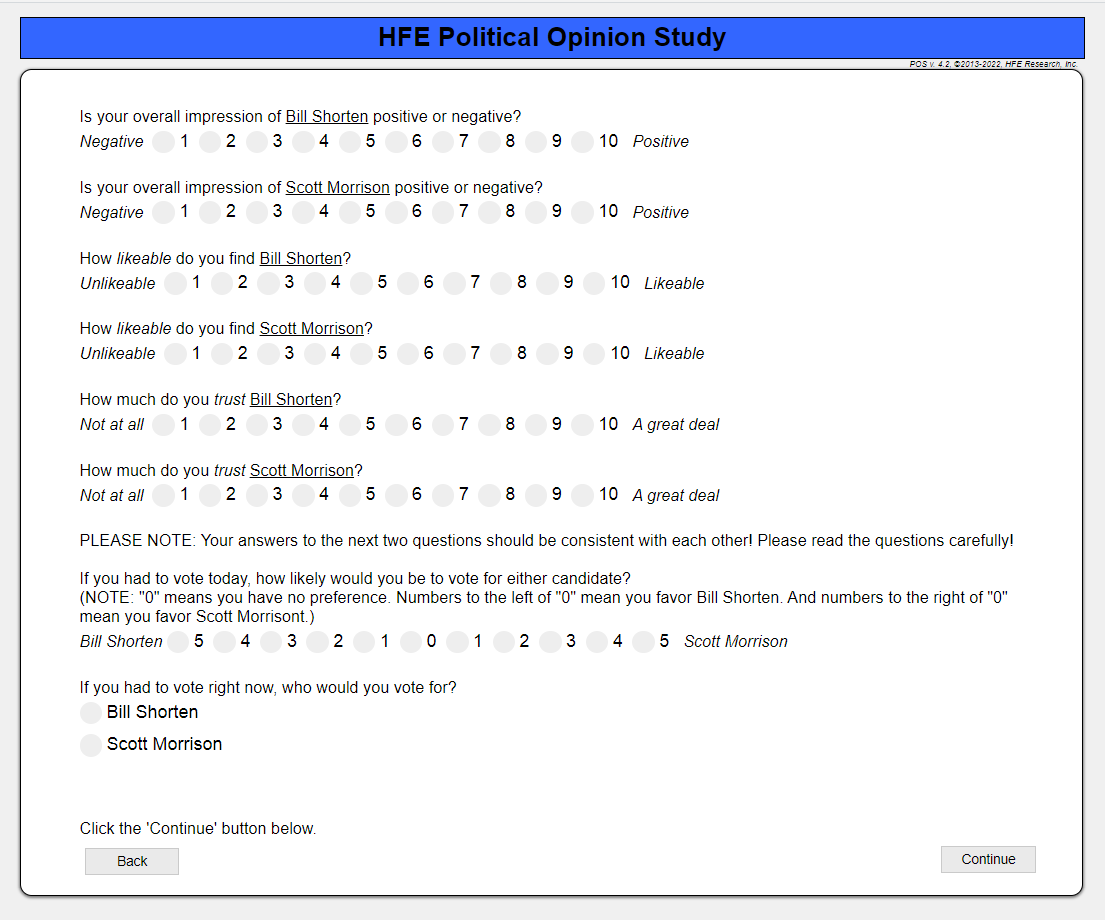
**S1 Fig. Investigation 2: Pre- and post-test opinion and voting questions.**

Supplement: S1 Fig — (DOCX) [file pone.0309897.s014.docx]

**
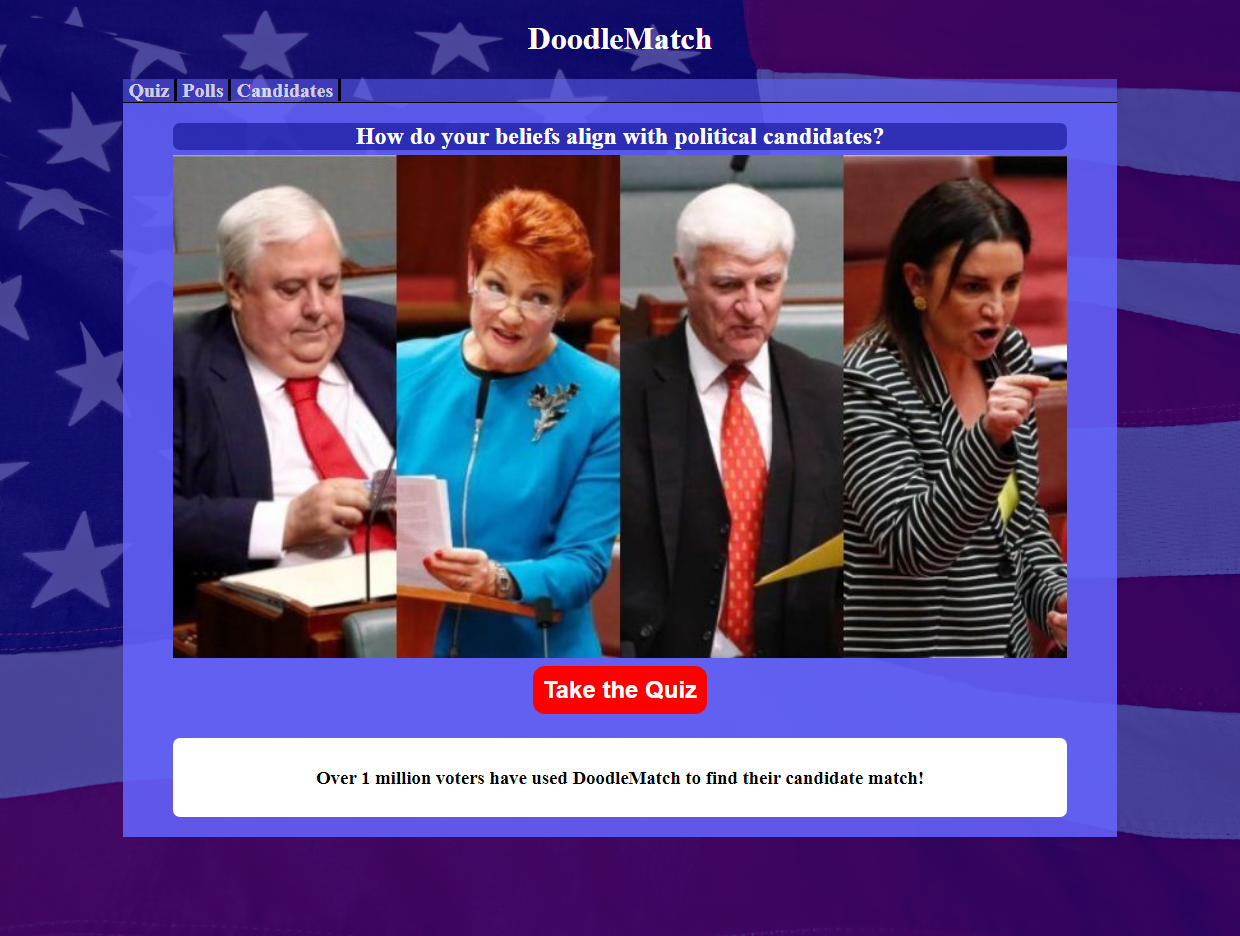
S2 Fig. DoodleMatch home page.**

Supplement: S2 Fig — (DOCX) [file pone.0309897.s015.docx]

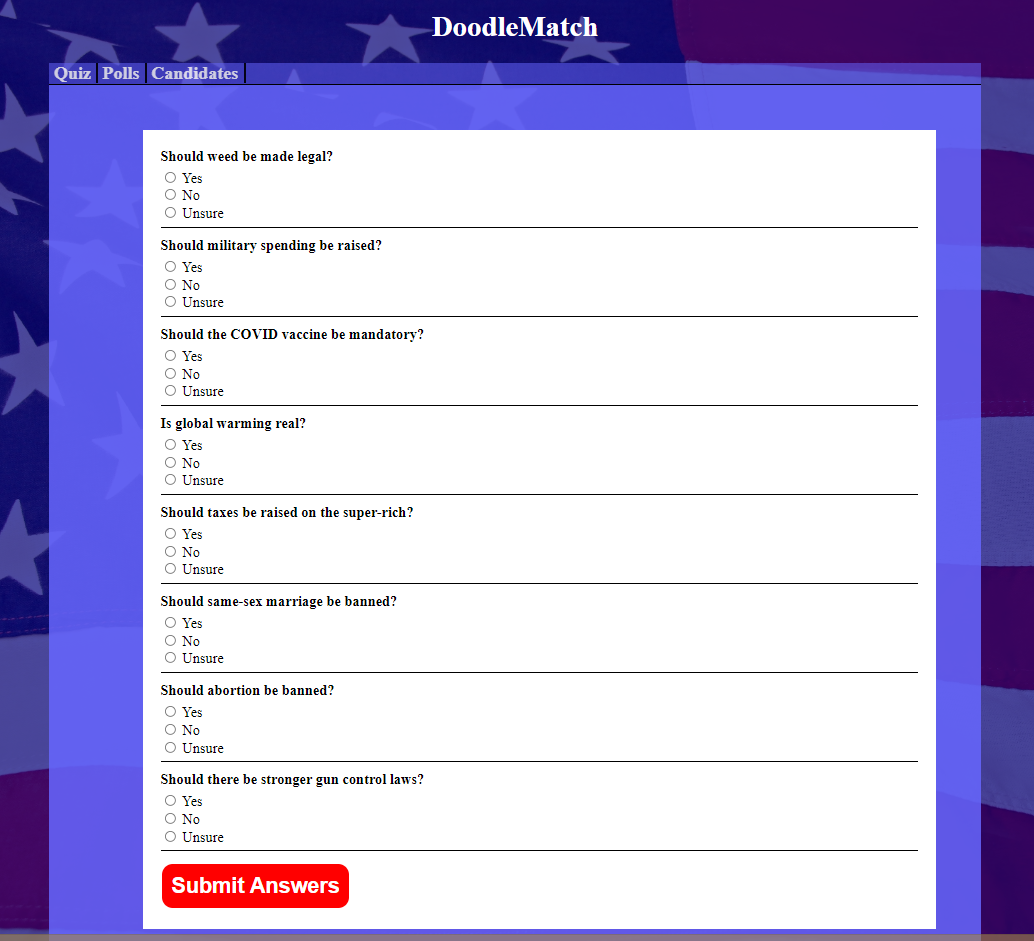
**S3 Fig. Investigation 2: 8-question, high readability quiz.**

Supplement: S3 Fig — (DOCX) [file pone.0309897.s016.docx]

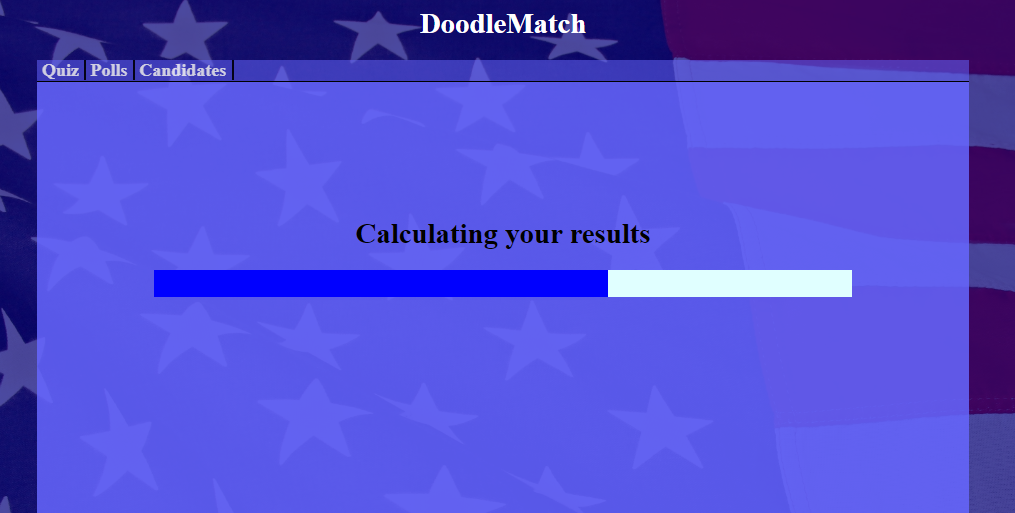
**S4 Fig. Investigation 2: Quiz result calculation bar.**

Supplement: S4 Fig — (DOCX) [file pone.0309897.s017.docx]

**
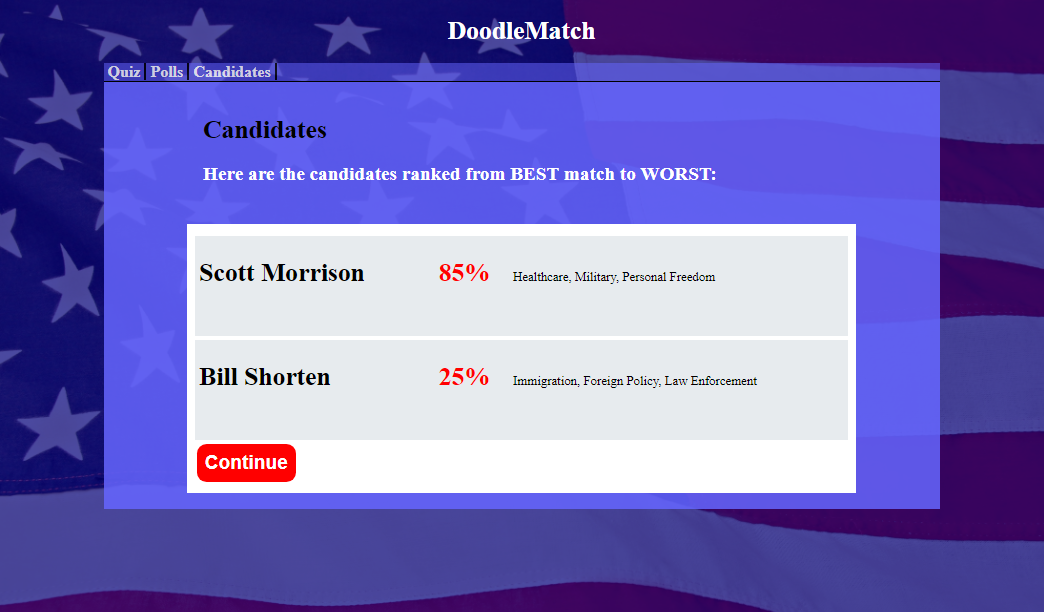
S5 Fig. DoodleMatch results page: Scott Morrison recommendation.**

Supplement: S5 Fig — (DOCX) [file pone.0309897.s018.docx]

**
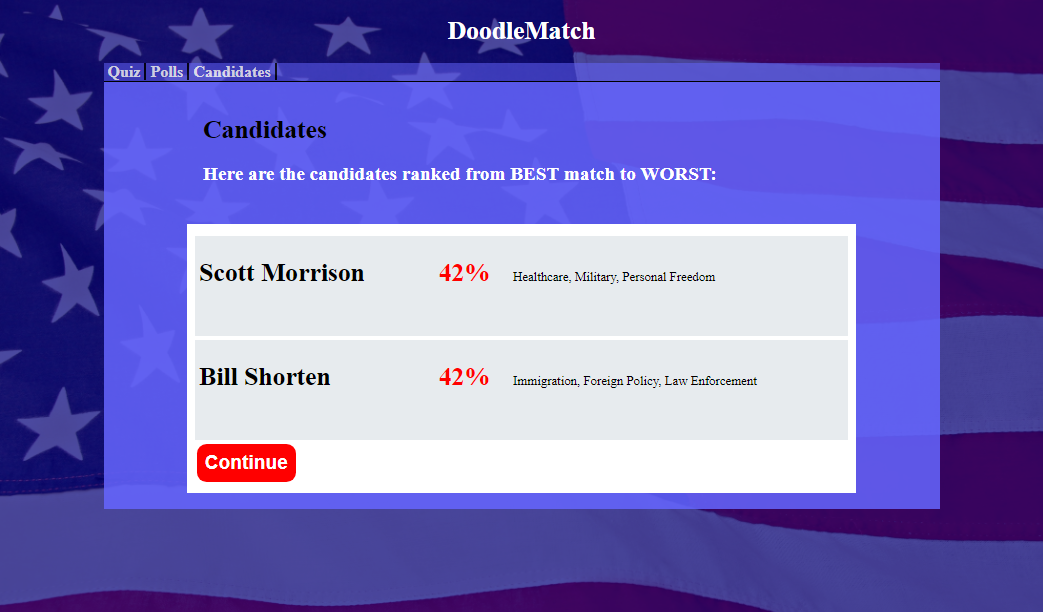
S6 Fig. DoodleMatch results page: Neutral group recommendation.**

Supplement: S6 Fig — (DOCX) [file pone.0309897.s019.docx]

**
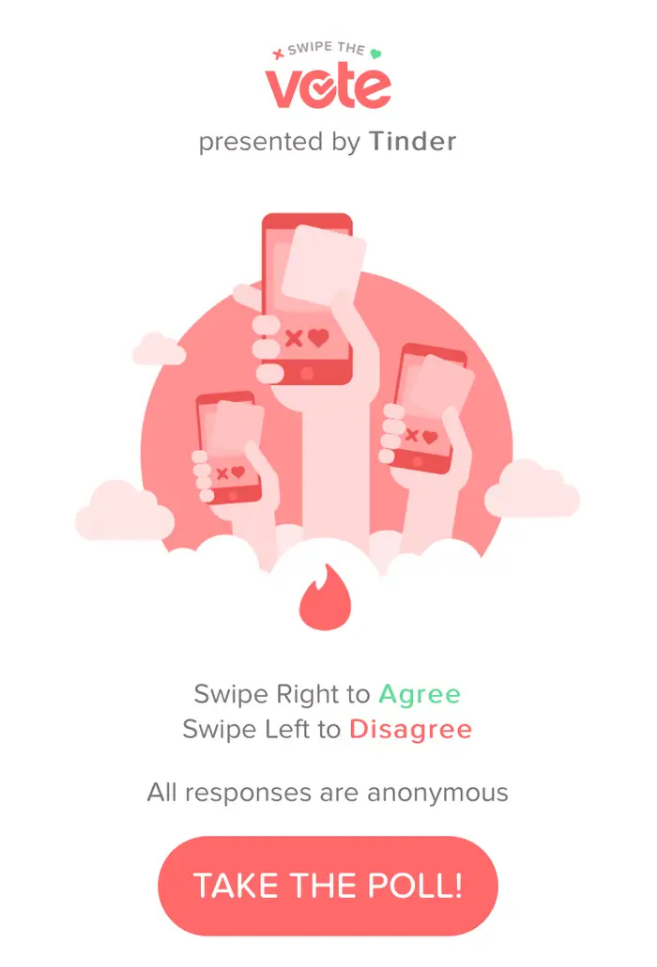
**

**S7 Fig. Tinder’s Swipe-the-Vote feature home page from March 23rd, 2016.**

Supplement: S7 Fig — (DOCX) [file pone.0309897.s020.docx]
