# Supplementary material for: The “opinion matching effect” (OME): A subtle but powerful new form of influence that is apparently being used on the internet
Source: PLoS One. 2024 Sep 12;19(9):e0309897. doi: 10.1371/journal.pone.0309897 (PMC11392280; doi:10.1371/journal.pone.0309897)
Supplement: S1 Table — (DOCX) [file pone.0309897.s021.docx]

**S1 Table. Demographic characteristics in Investigation 2 by quiz group.**

|  | **Group 1**  **(*n* = 197)** | **Group 2**  **(*n* = 197)** | **Group 3**  **(*n* = 187)** | **Group 4**  **(*n* = 192)** |
| --- | --- | --- | --- | --- |
| **Mean Age (*SD*)** | 37.6 (12.6) | 40.2 (13.1) | 40.8 (13.1) | 40.5 (12.6) |
| **Gender (%)** |  |  |  |  |
| **Male** | 86 (43.7) | 74 (37.6) | 92 (49.2) | 84 (43.8) |
| **Female** | 109 (55.3) | 123 (62.4) | 95 (50.8) | 107 (55.7) |
| **Other** | 0 (0.0) | 0 (0.0) | 0 (0.0) | 0 (0.0) |
| **Unknown** | 2 (1.0) | 0 (0.0) | 0 (0.0) | 0 (0.5) |
| **Political View (%)** |  |  |  | |
| **Conservative** | 35 (17.8) | 45 (22.8) | 45 (24.1) | 38 (19.8) |
| **Liberal** | 97 (49.2) | 88 (44.7) | 84 (44.9) | 103 (53.6) |
| **Moderate** | 57 (28.9) | 54 (27.4) | 52 (27.8) | 45 (23.4) |
| **None** | 6 (3.0) | 9 (4.6) | 5 (2.7) | 4 (2.1) |
| **Other** | 2 (1.0) | 1 (0.5) | 1 (0.5) | 2 (1.0) |
| **Unknown** | 0 (0.0) | 0 (0.0) | 0 (0.0) | 0 (0.0) |
| **Voter Status (%)** |  |  |  |  |
| **Decided** | 0 (0.0) | 0 (0.0) | 0 (0.0) | 0 (0.0) |
| **Undecided** | 197 (100) | 197 (100) | 187 (100) | 192 (100) |
| **Unknown** | 0 (0.0) | 0 (0.0) | 0 (0.0) | 0 (0.0) |
| **Fluency (*SD*)** | 9.9 (0.4) | 10.0 (0.1) | 10.0 (0.1) | 10.0 (0.3) |
