# Supplementary material for: The “opinion matching effect” (OME): A subtle but powerful new form of influence that is apparently being used on the internet
Source: PLoS One. 2024 Sep 12;19(9):e0309897. doi: 10.1371/journal.pone.0309897 (PMC11392280; doi:10.1371/journal.pone.0309897)
Supplement: S2 Table — (DOCX) [file pone.0309897.s022.docx]

**S2 Table. Investigation 2: Demographic analysis by educational attainment.**

| **Condition** |  | ***n*** | **VMP (%)** | **Mean Score Shift (SD)** |
| --- | --- | --- | --- | --- |
| **Bias Groups** | **≥ Bachelors** | 328 | 77.7 | 2.53 (2.64) |
|  | **< Bachelors** | 182 | 71.6 | 2.55 (2.52) |
|  | **Change (%)** | - | -7.9 | +0.8 |
|  | **Statistic** | *-* | *z* = 1.54 | t(508) = -0.09 |
|  | ***p*** | - | = 0.13 NS | = 0.46 NS |
