# Supplementary material for: The “opinion matching effect” (OME): A subtle but powerful new form of influence that is apparently being used on the internet
Source: PLoS One. 2024 Sep 12;19(9):e0309897. doi: 10.1371/journal.pone.0309897 (PMC11392280; doi:10.1371/journal.pone.0309897)
Supplement: S3 Table — (DOCX) [file pone.0309897.s023.docx]

**S3 Table. Investigation 2: Demographic analysis by gender.**

| **Condition** |  | ***n*** | **VMP (%)** | **Mean Score Shift (SD)** |
| --- | --- | --- | --- | --- |
| **Bias Groups** | **Male** | 222 | 74.3 | 2.44 (2.47) |
|  | **Female** | 287 | 75.7 | 2.61 (2.69) |
|  | **Change (%)** | - | +1.8 | +7.0 |
|  | **Statistic** | - | z = -0.36 | t(507) = -0.76 |
|  | ***p*** | - | = 0.72 NS | = 0.22 NS |
