# Supplementary material for: The “opinion matching effect” (OME): A subtle but powerful new form of influence that is apparently being used on the internet
Source: PLoS One. 2024 Sep 12;19(9):e0309897. doi: 10.1371/journal.pone.0309897 (PMC11392280; doi:10.1371/journal.pone.0309897)
Supplement: S4 Table — (DOCX) [file pone.0309897.s024.docx]

**S4 Table. Investigation 2: Demographic analysis by age.**

| **Condition** |  | ***n*** | **VMP (%)** | **Mean Score Shift (SD)** |
| --- | --- | --- | --- | --- |
| **Bias Groups** | **≥ 33** | 338 | 67.8 | 2.29 (2.55) |
|  | **< 33** | 172 | 92.6 | 3.03 (2.63) |
|  | **Change (%)** | - | +36.6 | +32.3 |
|  | **Statistic** | - | z = -6.21 | t(508) = -3.09 |
|  | ***p*** | - | < 0.001 | = 0.001 |
