# Supplementary material for: The “opinion matching effect” (OME): A subtle but powerful new form of influence that is apparently being used on the internet
Source: PLoS One. 2024 Sep 12;19(9):e0309897. doi: 10.1371/journal.pone.0309897 (PMC11392280; doi:10.1371/journal.pone.0309897)
Supplement: S5 Table — (DOCX) [file pone.0309897.s025.docx]

**S5 Table. Investigation 2: Demographic analysis by race/ethnicity.**

| **Condition** |  | ***n*** | **VMP (%)** | **Mean Score Shift (SD)** |
| --- | --- | --- | --- | --- |
| **Bias Groups** | **White** | 400 | 84.0 | 2.62 (2.56) |
|  | **Non-White** | 110 | 50.7 | 2.25 (2.70) |
|  | **Change (%)** | - | -39.6 | -14.1 |
|  | **Statistic** | - | z = 7.33 | t(508) = 1.31 |
|  | ***p*** | - | < 0.001 | = 0.10 NS |
