# Supplementary material for: The “opinion matching effect” (OME): A subtle but powerful new form of influence that is apparently being used on the internet
Source: PLoS One. 2024 Sep 12;19(9):e0309897. doi: 10.1371/journal.pone.0309897 (PMC11392280; doi:10.1371/journal.pone.0309897)
Supplement: S6 Table — (DOCX) [file pone.0309897.s026.docx]

**S6 Table. Investigation 2: Pre- and post-quiz mean voting preferences on 11-point scale for neutral groups by quiz group.**

| **Group** | **Neutral Groups *n*** | **Pre** | **Post** | **Diff** | ***z***^†^ | ***p*** |
| --- | --- | --- | --- | --- | --- | --- |
| **1.** 8 questions, high readability | 68 | 0.47 (2.70) | 0.69 (2.14) | 0.22 | -1.124 | 0.261 NS |
| **2.** 8 questions, low readability | 65 | 0.42 (2.49) | 0.40 (2.28) | -0.02 | -0.408 | 0.683 NS |
| **3.** 16 questions, high readability | 66 | 0.02 (2.59) | -0.09 (2.55) | -0.11 | -1.051 | 0.293 NS |
| **4.** 16 questions, low readability | 64 | -0.16 (2.85) | 0.05 (2.80) | 0.21 | -1.467 | 0.142 NS |
| **Total** | 263 | 0.19 (2.66) | 0.27 (2.45) | 0.08 | -0.642 | 0.521 NS |

^†^*z* values represent Wilcoxon signed ranks test comparing pre- and post-manipulation ratings on the 11-point scale.
