# Supplementary material for: The “opinion matching effect” (OME): A subtle but powerful new form of influence that is apparently being used on the internet
Source: PLoS One. 2024 Sep 12;19(9):e0309897. doi: 10.1371/journal.pone.0309897 (PMC11392280; doi:10.1371/journal.pone.0309897)
Supplement: S7 Table — (DOCX) [file pone.0309897.s027.docx]

**S7 Table. Investigation 2: ANOVA of opinion shifts (in the bias groups combined) for two factors: quiz length and readability.**

| **Opinion** | **Effect** | **Sum of Squares** | ***df*** | ***F*** | ***p*** |
| --- | --- | --- | --- | --- | --- |
| Impression | Quiz Length | 1.344 | 1 | 0.607 | 0.436 NS |
|  | Readability | 0.354 | 1 | 0.160 | 0.690 NS |
|  | Quiz Length × Readability | 0.012 | 1 | 0.005 | 0.941 NS |
| Trust | Quiz Length | 0.114 | 1 | 0.062 | 0.804 NS |
|  | Readability | 1.401 | 1 | 0.760 | 0.384 NS |
|  | Quiz Length × Readability | 1.679 | 1 | 0.911 | 0.340 NS |
| Likeability | Quiz Length | 3.529 | 1 | 1.661 | 0.198 NS |
|  | Readability | 4.009 | 1 | 1.887 | 0.170 NS |
|  | Quiz Length × Readability | 3.025 | 1 | 1.432 | 0.233 NS |
